# Drp1/Fis1 interaction mediates mitochondrial dysfunction, bioenergetic failure and cognitive decline in Alzheimer's disease

**DOI:** 10.18632/oncotarget.23640

**Published:** 2017-12-22

**Authors:** Amit U. Joshi, Nay L. Saw, Mehrdad Shamloo, Daria Mochly-Rosen

**Affiliations:** ^1^ Department of Chemical and Systems Biology, Stanford University School of Medicine, Stanford, CA 94305, USA; ^2^ Behavioral and Functional Neuroscience Laboratory, Department of Neurosurgery, Stanford University School of Medicine, Stanford, CA 94305, USA

**Keywords:** mitochondrial dysfunction, Alzheimer’s disease, Drp1, P110, patient-derived fibroblasts

## Abstract

Mitochondrial dynamics, involving a balance between fusion and fission, regulates mitochondrial quality and number. Increasing evidence suggests that dysfunctional mitochondria play a role in Alzheimer's disease (AD). We observed that Drp1 interaction with one of the adaptors, Fis1, is significantly increased in Aβ-treated neurons and AD patient-derived fibroblasts. P110, a seven-amino acid peptide, which specifically inhibits Drp1/Fis1 interaction without affecting the interaction of Drp1 with its other adaptors, attenuated Aβ_42_-induced mitochondrial recruitment of Drp1 and prevented mitochondrial structural and functional dysfunction in cultured neurons, in cells expressing mutant amyloid precursor protein (KM670/671NL), and in five different AD patient-derived fibroblasts. Importantly, sustained P110 treatment significantly improved behavioral deficits, and reduced Aβ accumulation, energetic failure and oxidative stress in the brain of the AD mouse model, 5XFAD. This suggests that Drp1/Fis1 interaction and excessive mitochondrial fission greatly contribute to Aβ-mediated and AD-related neuropathology and cognitive decline. Therefore, inhibiting excessive Drp1/Fis1-mediated mitochondrial fission may benefit AD patients.

## INTRODUCTION

Alzheimer’s disease (AD) is a late onset, progressive disease, characterized by neurodegeneration and impaired cognitive functions [1]. As the lifespan of humans increases, AD is becoming more prevalent disease. A characteristic feature of AD is the presence of extracellular amyloid plaques composed of the amyloid β peptides (mainly Aβ_40_ and Aβ_42_) in the brain. The vast majority of AD is sporadic (SAD), but mutations in the amyloid precursor protein (APP) and in presenilin-1 (PSEN1) and -2 (PSEN2), which are components of the γ-secretase complex that processes APP to produce amyloid-β (Aβ), have been identified in familial forms of AD (FAD), which have an earlier age of onset [2–4].

Mitochondria are essential organelles involved in oxidative phosphorylation, ATP production, metabolism of some neurotransmitters, amino acids and nucleotides, calcium homeostasis, reactive oxygen species management and programmed cell death [5–7]. Mitochondria are highly dynamic organelles that are reshaped by opposing processes of fusion and fission. Mitochondrial fission is mediated by dynamin-related protein 1 (Drp1), a large GTPase, that is recruited to the outer mitochondrial membrane (OMM) from the cytosol by several mitochondrial outer membrane protein adaptors, including fission 1 (Fis1), mitochondrial fission factor (Mff), as well as mitochondrial dynamics protein (MiD) of 49 and 51 [8–10]. Mitochondrial fragmentation (a pathological process) is caused by excessive mitochondrial fission and/or by reduced mitochondrial fusion [11, 12]. Both structural and functional abnormalities of mitochondria in AD have been observed in models of AD and in patients [2–4, 13–17]. These structural abnormalities are associated with an imbalance in proteins that control mitochondrial shape, size and number, increased Drp1 and Fis1 levels, and decrease in the levels of mitofusin 1 and 2 (Mfn1, Mfn2) and optic atrophy 1 (Opa1) [18–21]. Consistent with the above data, mitochondrial fission-linked GTPase activity is also significantly elevated in brain tissues from APP and APP/PS1 AD mouse models and in postmortem frontal cortex tissue from AD patients [22, 23]. The increase in mitochondrial fission proteins and decrease in mitochondrial fusion proteins likely results in increased mitochondrial structural damage seen in brains of AD patients; this, in turn, may contribute to disease progression as damaged mitochondria not only produce less ATP, but they also generate more damaging reactive oxygen species (ROS) that can propagate cytotoxicity of neighboring cells [1, 24]. Aβ oligomers induce mitochondrial fragmentation, and inhibition of their formation suppresses mitochondrial dysfunction and neuronal cell death [18, 23, 25–29]. Transmission electron microscopy of neurons treated with Aβ revealed a significant increase in mitochondrial fission [23, 27]. Thus, Aβ appears to induce excessive mitochondrial fission and dysfunction and neurodegeneration. Similarly, caspase-cleaved tau expressions in cortical neurons, another AD-associated phenotype, also leads to fragmented mitochondria [30]. Current treatments for AD do not directly target mitochondrial health. Cholinesterase inhibitors (donepezil, rivastigmine, and galantamine) and N-methyl-d-aspartate receptor antagonist (memantine) that are aimed to counteract imbalance in neurotransmission without affecting neurodegeneration per se are the only FDA-approved drugs for AD treatment, and both show only modest efficacy [31].

Recently, the effect of Drp1 inhibition using Mdivi-1 [32] on Aβ-mediated mitochondrial dysfunction and AD-associated neuropathology in cultured neurons and APP/PS1 double-transgenic AD mice has been investigated wherein the authors found pronounced improvement in both models tested [33]. However, while Mdivi-1 exerts protection of cardiovascular cells and neurons, this small molecule may be toxic to some cells, thus it might not be clinically beneficial in a chronic disease such as AD [34]. Importantly, a recent study provides strong evidence that in contrast to the original observation [32], Mdivi-1 does not inhibit Drp1 activity or mitochondrial fission; rather Mdivi-1 is a mitochondrial Complex-1 inhibitor with the ability to inhibit pathological ROS production [35]. Therefore, the benefit of selective inhibition of pathological fission in AD models has not yet been truly assessed.

In this study, we focused on inhibiting Drp1/Fis1 interaction using P110, a selective short inhibitory peptide of Drp1/Fis1 interaction that was developed in our lab [36]. The benefit of P110 treatment as an inhibitor of excessive (pathological) mitochondrial fission was determined using human neuroblastoma cell line SH-SY5Y cells treated with Aβ_42_ to induce Aβ-induced cytotoxicity, cells expressing a APP KM670/671NL double mutant, five different AD patient-derived fibroblasts and 5XFAD mouse model.

## RESULTS

### Drp1/Fis1 interaction increases during oligomeric Aβ_42_ (Aβ_42_) injury

**Figure 1:**
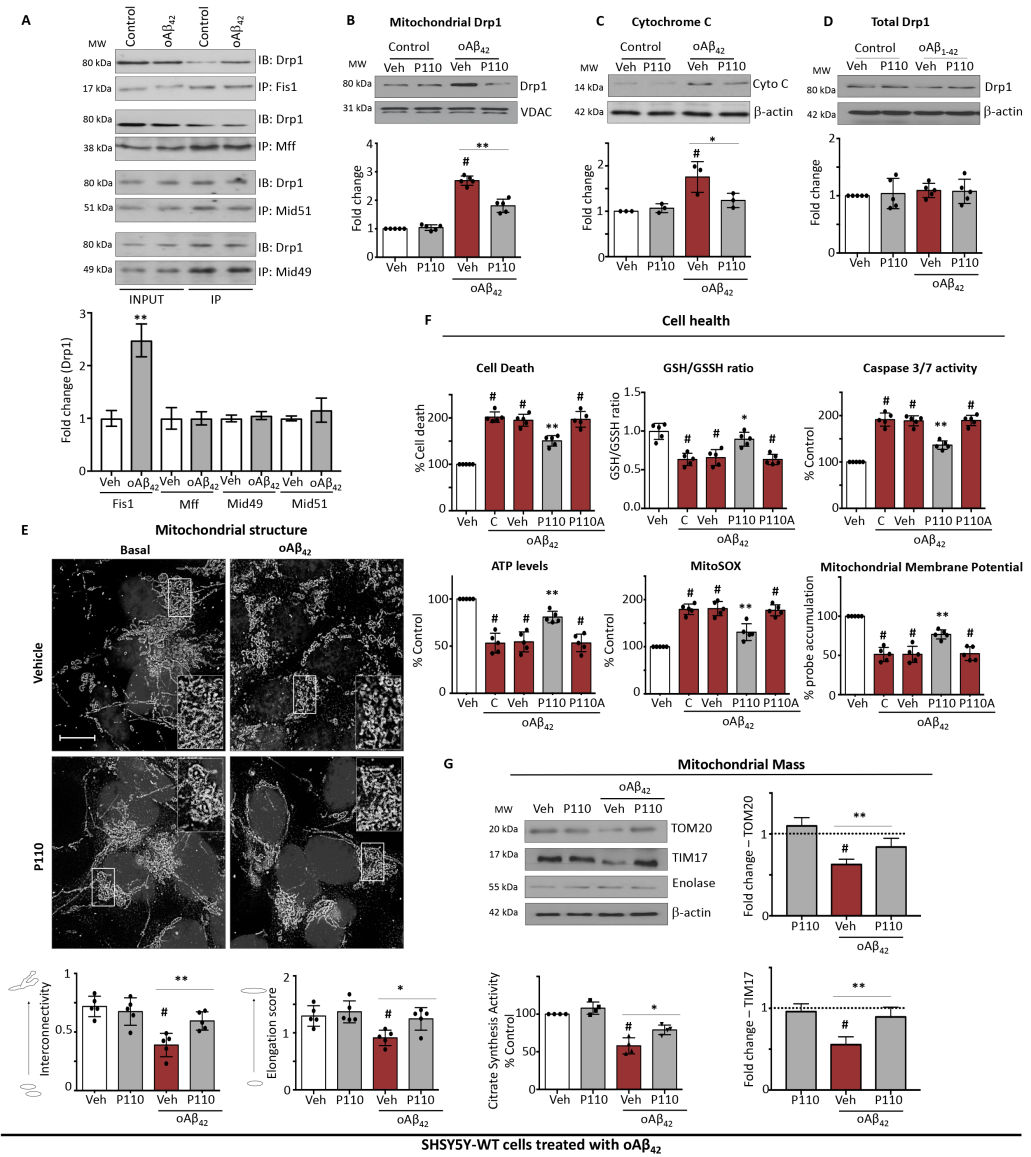
P110 reduces Aβ_42_ injury and subsequent mitochondrial defects in SH-SY5Y cells (**A**) Western blot analysis of immunoprecipitated Drp1 with its adaptors. *n =* 3. (**B**) Western blot showing Drp1 association with mitochondria-enriched fractions. VDAC, a mitochondrial membrane protein, was used as a loading control. Protein levels were quantified and represented as fold-change of vehicle treated control *n =* 5. (**C**) Western blot showing cytochrome c release into the cytosolic fraction; β-actin was used as loading controls. Protein levels were quantified and represented as fold-change of vehicle treated control *n =* 3. (**D**) Western blot of Drp1 in the total lysate; β-actin was used as loading controls. Protein levels were quantified and represented as fold-change of vehicle treated control *n =* 5. (**E**) Microscopy of stained SH-SY5Y cells with anti-TOM20 (a marker of mitochondria, 1:500 dilution) following incubation with Aβ_42_ for 24 hours. Scale bar: 5 µM. Inserts show enlarged areas of the white boxes. Below: Quantification of mitochondrial interconnectivity and mitochondrial elongation in SH-SY5Y cells following incubation with Aβ_42_. At least 150 cells per condition were counted. *n =* 5. (**F**) Cell death was measured using ApoTox-Glo™ after 24 hours of treatment as indicated. GSH/GSSG ratio was measured using GSH/GSSG-Glo™ 2 hours after adding oAβ_1-42_ (5 µM). Caspase 3/7 activity was measured using ApoTox-Glo™ after 24 hours of treatment. ATP levels were measured from using Mitochondrial ToxGlo™ after 24 hours of treatment. Mitochondrial superoxide production was determined using the mitochondrial superoxide indicator MitoSOX™ Red. Nuclei were stained with Hoechst (blue). Mitochondrial membrane potential was determined using JC-1 dye. *n =* 5. (**G**) Western blot of TOM20 and TIM17 as markers of mitochondrial mass in the total lysate; β-actin was used as loading controls. Protein levels were quantified and represented as fold-change of vehicle treated control *n =* 3. The activity of citrate synthase was also assayed using total cell lysates to determine the mitochondrial mass. Data information: Mean, standard deviation, and *P-*values are shown. ^#^*P <* 0.05 versus controls. ^*^P < 0.05; ^**^P < 0.01; ^***^P < 0.001; ^****^P < 0.0001. One-way ANOVA and Tukey’s post hoc test. C, control.

The cytosolic Drp1 has at least four different mitochondrial adaptors: Mff, Fis1, Mid49 and Mid51 [8]. We first demonstrated that Drp1 interaction with Fis1 is induced by Aβ_42_, whereas no change was observed with its interaction with other adaptors in SHSY5Y cells treated with 5 µM Aβ_42_ for 24 hours (Figure 1A). Since Drp1 recruitment from the cytosol to the outer mitochondrial membrane is a hallmark of activated mitochondrial fission, we then determined whether blocking Drp1/Fis1 interaction by P110 treatment, is sufficient to reduce Drp1-mitochondrial association. As compared with controls, Drp1 association with the mitochondria increased by 2.7-fold (Figure 1B) and mitochondrial dysfunction was evidenced by increased cytochrome c release (a marker of apoptosis) by 1.8 fold (Figure 1C) although total cellular levels of Drp1 remained unchanged throughout the Aβ_42_ treatment (Figure 1D). A 24 hour treatment with (1 µM) P110 greatly inhibited Drp1 association with the mitochondria in Aβ_42_ treated cells, resulting in decreased cytochrome c release (Figure 1B–1C), while showing no effect on the total Drp1 levels (Figure 1D).

Since Drp1 recruitment to the mitochondria is an essential step in triggering mitochondrial fission, we next determined the effect of Aβ_42_ on mitochondrial morphology in SHSY5Y cells by immunofluorescence, using the mitochondrial marker, TOM20, and determined changes in mitochondrial shape by calculating indices of mitochondrial interconnectivity and mitochondrial elongation. Excessive mitochondrial fragmentation was induced in cultures treated with Aβ_42_, as evidenced by small, round or dot-like staining patterns (Figure 1E). Aβ_42_ treatment decreased mitochondrial interconnectivity and mitochondrial elongation (Figure 1E), all features associated with increased mitochondrial fragmentation. P110 treatment greatly corrected these mitochondrial structure abnormalities in cells exposed to Aβ_42_, restoring mitochondrial interconnectivity and length (Figure 1E). P110-treatment correction of Aβ_42_-induced mitochondrial structural defects in SHSY5Y cells were associated with reduced cell toxicity relative to control/vehicle-treated or an inactive P110 analog, in which one amino acid was substituted with alanine [P110A; [36]], (P110A)-treated cells (Figure 1F). Reduced glutathione (GSH) is one of the most important scavengers of reactive oxygen species (ROS), and its ratio with oxidized glutathione (GSSG) is a marker of oxidative stress. Treatment with Aβ_42_ caused a decrease in the GSH/GSSG ratio (to 0.7 of control), which was corrected by P110 treatment (from 0.7 to 0.95; Figure 1F). In addition, the increase in the activity of the pro-apoptotic enzymes, caspase 3 and caspase 7, after Aβ_42_ (195 ± 24% of basal) was significantly reduced by P110 treatment (Figure 1F). As for cell toxicity above, both glutathione levels and caspase activation were unaffected by treatment with an inactive P110 analog as well as by treatment with vehicle control in Aβ_42_-treated SHSY5Y cells (Figure 1F).

Others and we have previously reported that Drp1-dependent mitochondrial fission impairment occurs during the early stage of mitochondrial dysfunction [13, 18, 36] and a drop in ATP levels have been observed both in *in vitro* and *in vivo* models of AD [37–39]. Aβ_42_ treatment of SHSY5Y cells also resulted in a 50 ± 10% reduction in ATP levels, and the levels significantly increased following P110 treatment (to 80 ± 10%; Figure 1F). Furthermore, the production of mitochondrial superoxide was greatly elevated in cells treated with Aβ_42_ as measured by MitoSOX, and this Aβ effect was also abolished by treatment with P110 (Figure 1F). Finally, Aβ_42_-induced 50% loss in the mitochondrial membrane potential, as measured by JC1 probe accumulation thereby possibly contributing to mitochondrial fragmentation. This observed effect was also significantly corrected by P110 treatment, but not by treatment with two control peptides (Figure 1F). (Note that given the protective effect of P110, there is a possible contribution of inhibition of mitochondrial fragmentation and the associated shape change to the increase in JC1 probe intensity; a contribution that is not easy to dissect from simple increase in membrane potential.)

Mitochondrial mass decrease was also observed in cell treated with Aβ_1–42_ as measured by the reduction in total levels of TOM20 and TIM17, markers of outer and inner mitochondrial membrane, respectively. P110 treatment greatly reduced this loss (Figure 1G). Citrate synthase is used as a common matrix enzyme marker, and citrate synthesis activity reflects mitochondrial mass [40, 41]. We found an ∼50% loss in mitochondrial mass in cells treated with Aβ_42_ and P110 treatment inhibited this Aβ_42_-induced loss (Figure 1G).

### P110 treatment prevented mitochondrial dysfunction in N2a cells expressing the “Swedish” APP mutant form, APPSwe

**Figure 2:**
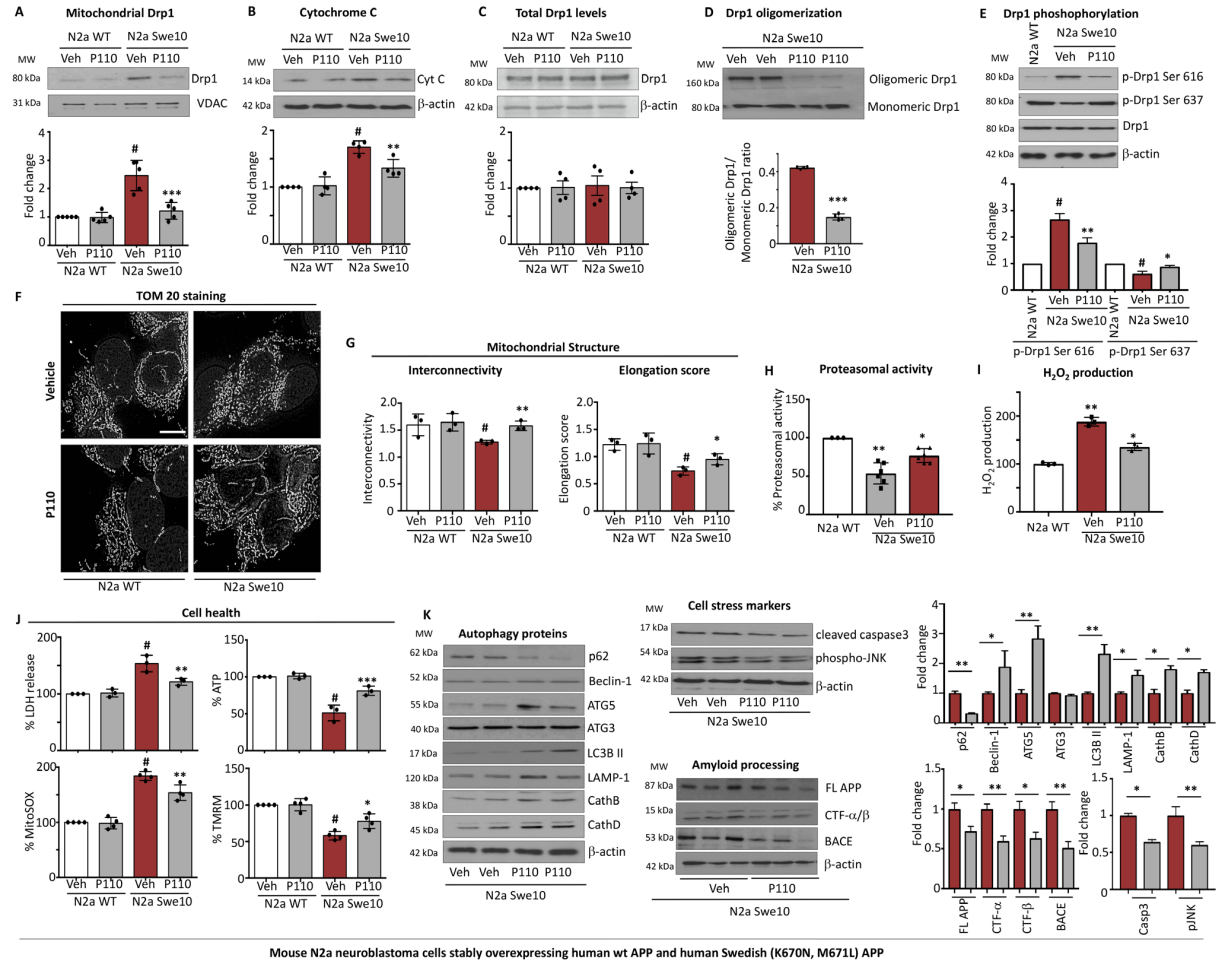
Treatment with P110 blocked increased Drp1 association with the mitochondria induced by Swe10 mutation and the associated mitochondrial damage in mouse N2a neuroblastoma cells (**A**) Western blot showing Drp1 association with mitochondria-enriched fractions, VDAC was used as loading control. Protein levels were quantified and represented as fold-change of vehicle treated control. *n =* 5. (**B**) Western blot showing cytosolic cytochrome c levels, β-actin was used as loading control. Protein levels were quantified and represented as fold-change of vehicle treated control. *n =* 4. (**C**) Western blot showing Drp1 levels in total lysate; β-actin was used as loading control. Protein levels were quantified and represented as fold-change of vehicle treated control. *n =* 4. (**D**) Drp1 oligomerization in total lysates was measured under native conditions. Protein levels were quantified and represented as ratio. *n =* 4. (**E**) Drp1 phosphorylation levels were examined in total lysate by immunoblotting using anti-phosphorylated-S616-Drp1 or -S637-Drp1 antibodies in the presence or absence of P110 (1 µM/48 hours); β-actin was used as a loading control. Protein levels were quantified and represented as fold-change of N2a-WT. *n =* 3. (**F**) Microscopy of stained N2a WT and N2a Swe10 cells with anti-TOM20 (a marker of mitochondria, 1:500 dilution) incubated in the presence or absence of P110 (1 µM) for 12 hours in serum free medium. Scale bar: 0.5 µm. *n =* 3. (**G**) Quantification of mitochondrial interconnectivity and elongation scores in N2a WT and N2a Swe10 cells. At least 200 cells per condition were counted. *n =* 3. (**H**) Chymotrypsin-like activity was measured using fluorogenic substrate; Suc-LLVY-AMC to measure proteasome activity in homogenates of N2a cells. Activity levels were quantified and represented as %-change of N2a WT *n =* 3. (**I**) Amplex red assay was used to quantitate H2O2 generation and represented represented as %-change of N2a WT *n =* 3. (**J**) Cytotoxicity levels were measured using ApoTox-Glo™ after 48 hours of treatment. ATP levels were measured using ApoTox-Glo™ after 48 hours of treatment. Mitochondrial superoxide production was determined using the mitochondrial superoxide indicator MitoSOX™ Red. Mitochondrial membrane potential was determined using JC-1 dye. (**K**) Levels of p62, Beclin-1, ATG5, ATG3, LC3BII, LAMP-1, CathB, CathD (measures of Autophagic flux), Cleaved caspase -3, phosho JNK (measures of cell stress) and FL-APP, CTF α/β, BACE-1 (measures of APP processing) in total fractions were measured by immunoblotting in N2a Swe10 cells in the presence or absence of P110 (1 µM/24 hours); β-actin were used as a loading controls. Protein levels were quantified and represented as fold-change of vehicle. *n =* 3. Data information: Mean, standard deviation, and *P*-values are shown. ^#^*P* < 0.05 versus controls. ^*^P < 0.05; ^**^P < 0.01; ^***^P < 0.001; ^****^P < 0.0001. One-way ANOVA and Tukey’s post hoc test. C, control.

We next examined the consequence of inhibiting Drp1/Fis1 interaction in another model of AD disease, neuroblastoma cells expressing a mutant form of APP (695), a mutation that is associated with FAS, found in a Swedish family (KM670/671NL). Cell culture studies show that this mutation increased the production and secretion of Aβ_40_ and Aβ_42_ [42, 43]. We observed that P110 treatment (48 hours; 1 µM) decreased mitochondrial association of Drp1 (Figure 2A) and cytochrome c release from the mitochondria into the cytosol in N2a cells expressing the double mutation (N2a Swe10) (Figure 2B) as compared to cells expressing WT APP (N2a WT). No changes in total Drp1 levels were observed under basal or P110-treated conditions (Figure 2C), but the oligomerization level of Drp1, a function of its activity, was significantly inhibited by P110 treatment (Figure 2D). The regulation of Drp1 by post-translational modifications is important for Drp1 translocation to mitochondria [44]. Phosphorylation of Drp1 at Ser-616 by cyclin-dependent kinase (CDK) 1/Cyclin B or CDK5 promotes mitochondrial fission, whereas de-phosphorylation of Drp1 at Ser-637 by calcineurin facilitates its translocation to mitochondria and subsequently increases mitochondrial fission [45, 46]. Therefore, a balance between Drp1 Ser-616/Ser-637 phosphorylation ratio reflects Drp1 activity. Western blot analysis of total protein lysates showed a significant increase in Drp1 phosphorylation at Ser-616 combined with a decrease in phosphorylation at Ser-637 in these cells (Figure 2E). These results indicate that Drp1 hyperactivation and phosphorylation occur in neurons expressing mutant APP and that treatment with P110 inhibits this hyperactivation.

As expected, the consequence of Drp1 hyperactivation and recruitment to the mitochondria in the N2a Swe10 cells is decreased mitochondrial elongation score (*P <* 0.01) and interconnectivity score (*P <* 0.05; Figure 2F, 2G), and P110 treatment greatly reduced these effects on mitochondrial morphology, improving mitochondrial interconnectivity (*P <* 0.01) as well as elongation (*P <* 0.05), without affecting these parameters in WT cells (Figure 2F, 2G).

Aβ_42_ is degraded by the proteasome and partial inhibition of proteasome activity leads to the formation of plaques, which contributes to neuropathology [47]. The N2a Swe10 cells show a decreased chymotrypsin-like proteasome activity and P110 treatment partially restored it (Figure 2H). Similarly, the increased oxidative stress as measured by production of hydrogen peroxide and cell death was significantly reduced by P110 (Figure 2I, 2J). On the bioenergetic level, the double APP mutation significantly reduced ATP levels confirming the toxic effects of this mutation, were also inhibited by P110 treatment (Figure 2J). Mitochondrial superoxide level elevation (Figure 2J) and a lower mitochondrial membrane potential (Figure 2J) were observed in N2a Swe10 cells when cultured under serum starvation conditions for 48 hours and P110 treatment (48 hours; 1 µM/ 24 hours) attenuated these dysfunctions (Figure 2J). Again, P110 treatment had no effect on any of these parameters in N2a WT cells (Figure 2J).

Recently, autophagy flux was observed to be progressively impeded resulting in deficient substrate clearance, and reflected by autolysosomal accumulation of LC3-II and SQSTM1/p62 and expansion of autolysosomal size and total area. We measured the levels of p62, Beclin-1, ATG3/5, LC3BII, LAMP-1, CathB and CathD as markers of autophagy. P110 treatment restored this imbalance, indicating the importance of mitochondrial health in autophagic flux (Figure 2K). Next, we investigated the levels of cell stress markers, cleaved caspase 3 and phospho-JNK and observed a reduction in their levels when cells were treated with P110 (Figure 2K). The first step in Aβ generation is cleavage of APP by the β-secretase BACE-1. After α- and β-cleavage, the carboxyl terminal fragments (CTFs) of APP, known as αCTF and βCTF, respectively, remain membrane-associated and is further cleaved by γ-secretase. We observed that improving mitochondrial health with P110 reduced the levels of these toxic processing proteins (Figure 2K).

### Inhibiting Drp1/Fis1 interaction with P110 treatment improved mitochondrial function in AD patient-derived fibroblasts

**Figure 3:**
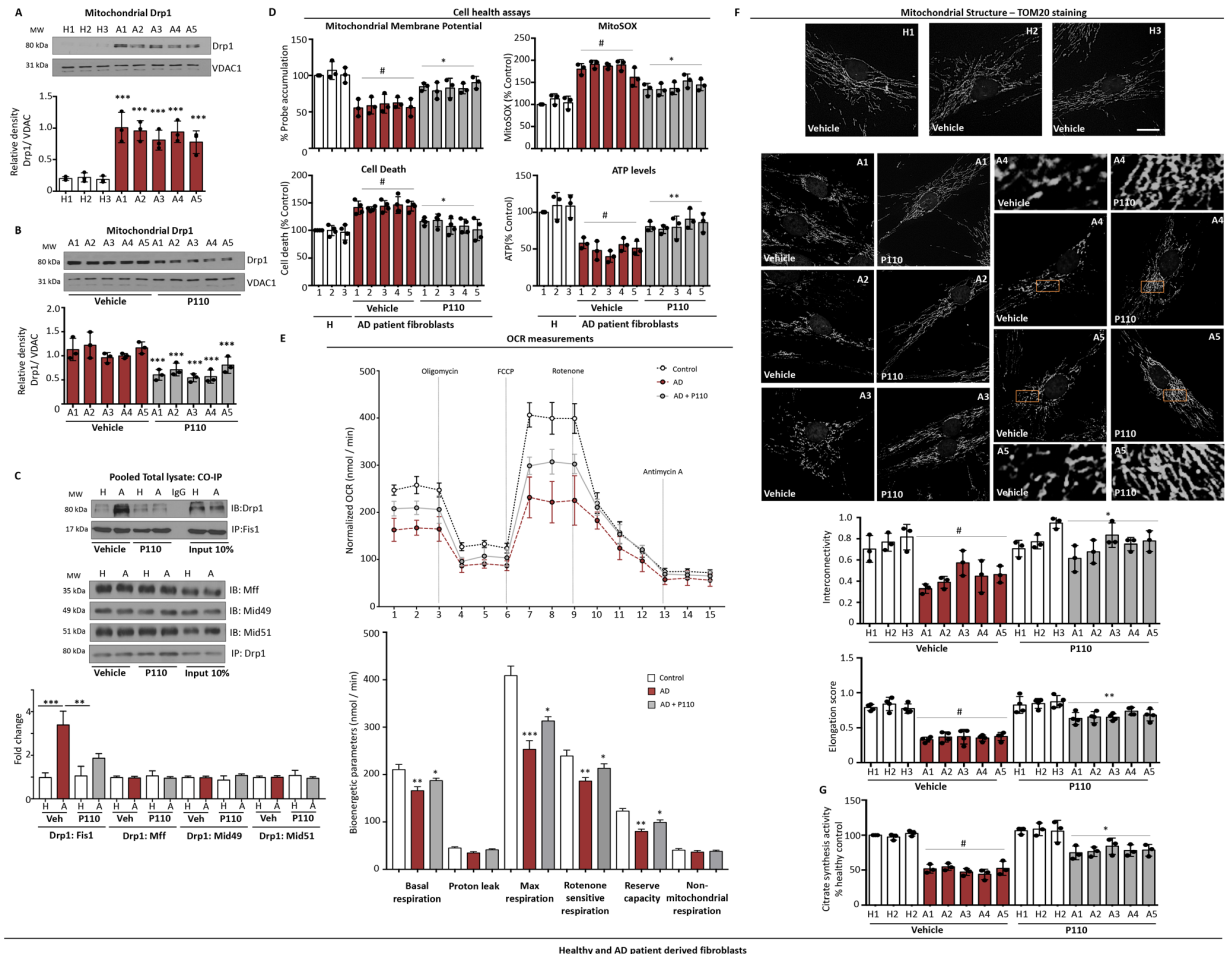
Drp1/Fis1 interaction leads to mitochondrial defects and bioenergetic failure in AD patient derived fibroblasts (**A**) Western blot showing Drp1 association with mitochondria-enriched fractions after 72 hours incubation in 1% FBS medium. VDAC was used as loading control. Protein levels were quantified and represented as ratio. *n =* 3. (**B**) Western blot showing Drp1 levels in mitochondria-enriched fractions after 72 hours incubation in the presence or absence of P110 in 1% FBS medium. VDAC was used as loading control. Protein levels were quantified and represented as ratio. *n =* 3. (**C**) AD patient-derived and healthy subject-derived fibroblasts were incubated in serum free medium in the presence or absence of P110 for 72 hours, pooled total cell lysates were subjected to immunoprecipitation (IP) with anti-Fis1 or anti-Drp1 and the immunoprecipitates were analyzed by immunoblotting (IB); *n =* 3. (**D**) Mitochondrial membrane potential was determined using JC-1 dye after 48 hours. Mitochondrial superoxide production was determined using the mitochondrial superoxide indicator MitoSOX^™^ after 48 hours. Cell death was measured using ApoTox-Glo™ after 72 hours. ATP levels were measured from using Mitochondrial ToxGlo^™^ after 72 hours; *n =* 3. (**E**) OCR of healthy controls and AD-patient derived fibroblasts was measured at the basal level and then after sequential treatment with DMEM/200 mM Eto followed by 1 mM Oligo, 1.5 mM FCCP, 0.1 mM Rot and 1 mM Ant A using the XF-96 Seahorse system. The assay is representative of two independent experiments, run in triplicates. Bioenergetic parameters were quantified and are represented below. (**F**) Microscopy of stained healthy control fibroblasts H1: AG07123 from 62 year old male, H2:AG04146 from 57 year-old male and H3: HDFA and AD-patient derived fibroblasts A1: AG04402 from 47 year old male; Familial-APOE, A2:AG07377 from 60-year-old male; Sporadic AD; A3:AG06840 from 56 year-old male; Familial- PSEN1; A4:AG09908 from 81 year old male; Familial-PSEN2; A5:AG05810 from 79 year-old female; Familial-APOE with anti-TOM20 (a marker of mitochondria, 1:500 dilution) incubated in the presence or absence of P110 (1 µM) for 72 hours in serum free medium. Panels show enlarged areas of the boxes. Scale bar: 0.5 µm. Below, quantification of mitochondrial interconnectivity and elongation score in healthy control fibroblasts and AD-patient derived fibroblasts. (**G**) The activity of citrate synthase was determined in Healthy controls and AD-patient derived fibroblasts using total cell lysates to indicate the mitochondrial mass. Data information: Mean, standard deviation, and *P-*values are shown. ^#^*P* < 0.05 versus controls. ^*^P < 0.05; ^**^P < 0.01; ^***^P < 0.001; ^****^P < 0.0001. One-way ANOVA and Tukey’s post hoc test. C, control.

Patient-derived fibroblasts have been employed as an *in vitro* model for AD [48–51] and, a fibroblast biomarker profile was proposed to identify accurately AD patients for therapeutic intervention [51]. We used the following patient-derived fibroblasts: one from a patient with a sporadic AD (A1), two from patients with APOE4 mutation (A2 and A3), another from a patient with PSEN1 (A4), and one from a patient with PSEN2 (A5). We first determined Drp1 localization on the mitochondria in these dermal fibroblasts. When cultured in 1% serum-containing media for 72 hours, we observed more than a three-fold higher Drp1 association with the mitochondria relative to fibroblasts derived from unaffected control subjects (Figure 3A). This increased Drp1 association with the mitochondria was significantly reduced by treating the AD patient-derived fibroblasts with 1 µM P110 (1 µM every 24 hours for 3 days; Figure 3B). Culturing under serum-starved conditions for 72 hours substantially increased the direct Drp1/Fis1 interaction in patient-derived fibroblasts relative to fibroblasts from a healthy control, as determined by co-immunoprecipitation and this increased Drp1/Fis1 association was abolished by P110 treatment (Figure 3C). Importantly, this effect was specific; association of Drp1 with Mff, Mid49 and Mid51 were not higher in AD cells and P110 treatment did not affect this interaction (Figure 3C). Significant reductions in mitochondrial membrane potential, through the decreased accumulation of JC1 probe (70 ± 10% of control cells; Figure 3D) and increased mitochondrial superoxide production, accumulation of MitoSOX probe (180 ± 20% of control cells; Figure 3D) observed in these AD patient-derived cells were also corrected with P110 treatment (*P <* 0.05). (Note that the same caveat related to the probe use, as indicated for Figure 1). Furthermore, increased cytotoxicity as well as the ∼50% reduction in ATP levels observed in the AD patient-derived fibroblasts were reversed with P110 treatment (*P <* 0.05; Figure 3D).

The increased Drp1 association with the mitochondria correlated with increased mitochondrial fragmentation in fibroblasts from AD patients with decreased mitochondrial interconnectivity and elongation scores (Figure 3E). There was also a ∼50% loss in mitochondrial mass, as measured by citrate synthesis activity and all were corrected by P110 treatment (Figure 3G). The bioenergetic properties of AD and control fibroblasts were assessed using the Extracellular Flux Analyzer. According to the OCR values, six bioenergetic parameters were also calculated and significant decrease was found in basal respiration, maximal respiration, rotenone-sensitive respiration, reserve capacity in AD fibroblasts. P110 treatment corrected these; no differences were observed in proton leak and non-mitochondrial respiration (Figure 3F). Taken together, we found that inhibition of mitochondrial excessive fission by inhibiting Drp1/Fis1 interaction with P110 treatment was beneficial when tested in fibroblasts of humans who have sporadic or familial forms of AD.

### Inhibition of Drp1/Fis1 interaction is sufficient to reduce cognitive decline in a 5XFAD mouse model

**Figure 4:**
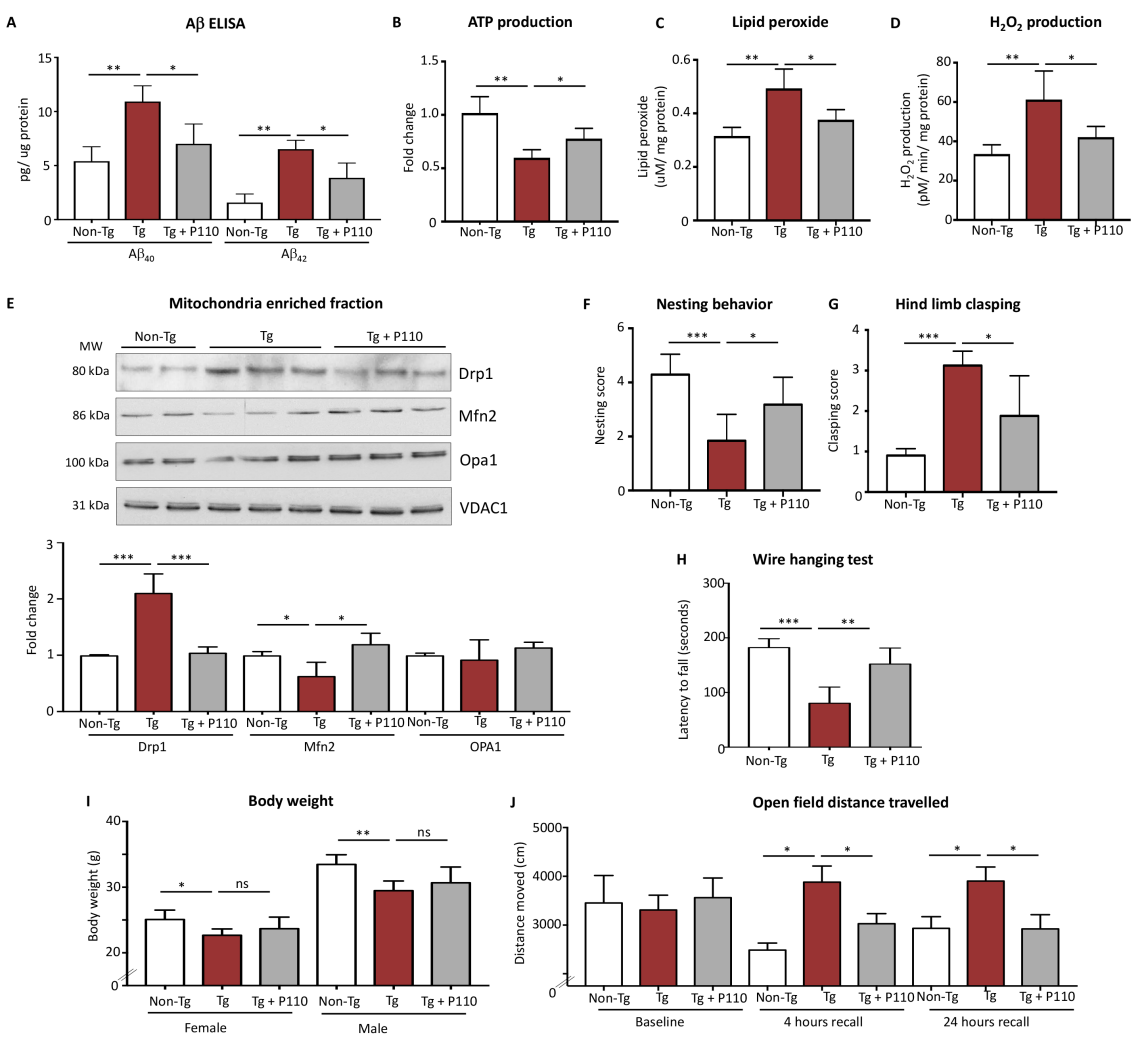
Inhibition of Drp1 association with Fis1 using P110 in the symptomatic phase improves survival and slows disease progression (**A**) Aβ_40_ and Aβ_42_ levels were examined via ELISA in 5xTg-AD mitochondrial fractions. *n =* 5. (**B**) The ATP colorimetric/fluorometric assay kit was used to measure ATP levels in freshly isolated whole brain mitochondria. *n =* 5. (**C**) Lipid peroxide levels were determined by the leucomethylene blue assay in whole brain mitochondria. *n =* 5. (**D**) Rates of hydrogen peroxide production were determined in state-4 respiration by Amplex-Red hydrogen peroxide assay from whole brain mitochondria. *n =* 5. (**E**) Western blot showing Drp1, Mfn2 and Opa1 mitochondria-enriched fractions from whole brains. VDAC was used as loading control. Protein levels were quantified and represented as fold change. *n =* 4–6. (**F**) Non-learnt behavior as assessed by nesting behavior in home change. *n =* 4–6 per group. (**G**) Hindlimb clasping scores, as assessed in the tail suspension test. *n =* 10 per group. (**H**) Latencies to fall in the context of a wire hang test. *n =* 10 per group. (**I**) Body weight measurements separated into sexes. *n =* 5 per group. (**J**) Distance travelled was measured in a 10 min open field test after 10 min of habituation. *n =* 10 per group. Data information: Mean, standard deviation, and *P*-values are shown. ^*^P < 0.05; ^**^P < 0.01; ^***^P < 0.001; ^****^P < 0.0001. One-way ANOVA and Tukey’s post hoc test.

To assess the benefit of sustained treatment with P110 *in vivo*, we used 5XFAD mouse model of AD. Three-month old mice were implanted with an Alzet pump under the skin in the upper back. The pump delivered P110 at 3 mg/day subcutaneously, pumps were replaced every month and the procedure was repeated for 3 months. We observed significant increases in both Aβ_40_ and Aβ_42_ in both the AD mitochondrial fractions compared with non-demented controls in 5XFAD (using ELISA) and P110 treatment resulted in a significant reduction in both (Figure 4A). At the end of the study (age of 6 months), ATP levels in whole brain lysate of the 5XFAD mice were ∼40% lower relative to WT mice and this loss in ATP levels was lessened by sustained treatment with P110 (Figure 4B). To determine mitochondrial oxidative load, we assessed the magnitude of lipid peroxidation and the rate of hydrogen peroxide production in mitochondria isolated from whole brain of 5XFAD at the age 6 months and found a significant increase in lipid peroxidation, which was reduced by P110 treatment (Figure 4C). The Amplex Red hydrogen peroxide assay was used to determine the rates of hydrogen peroxide production of mitochondria in both state 4 (in the absence of ADP) and state 3 (in the presence of ADP) respiration and as demonstrated by this test, there was a significant increase in the rate of state 4 hydrogen peroxide production in the 5XFAD transgenic mice as compared with age-matched non-transgenic mice, and this was almost completely abolished by P110 treatment (Figure 4D).

To validate that P110 treatment affected its target, we determined Drp1 association with the mitochondria in the three groups of mice. There was a 2-fold increase mitochondrial association of Drp1 in 5XFAD mice as compared with control mice, which was completely inhibited following sustained P110 treatment (Figure 4E). There was also a partial reduction in MFN2 protein levels, which was restored by P110 treatment, whereas no changes were observed in OPA1 levels in 5XFAD mice relative to WT regardless of P110 treatment (Figure 4E).

To estimate non-learned behaviors in the mice, which are meant to mimic neuropsychiatric and signs of cognitive losses in humans, nest-building behavior was monitored [52]. A marked decrease in nest building ability was observed in 5XFAD mice and this behavior was improved with sustained P110 treatment (Figure 4F). P110 treatment ameliorated also pathological hindlimb clasping, likely the result of deficient neuronal inhibitory processes in 5xFAD mice (Figure 4G). 5XFAD mice have been found to have motor defects when measured with wire hang test [53]. 5XFAD mice showed significant deficits, which were partially corrected with P110 treatment (Figure 4H). This improvement was unlikely due to change in body weight in P110-treated 5XFAD vs. control-treated 5XFAD mice (Figure 4I). Finally, when we used an open-field test to assess exploration behavior in a relatively large novel environment as compared to the activity chamber, there were no changes under baseline condition. However, when tested after 4 and 24 hours for a short-term and long-term recall, respectively, the 5XFAD mice did not reduce the wandering distance in the open field whereas with control mice did, suggesting impairment in memory (Figure 4J). Importantly, the wondering distance of P110-treated 5XFAD mice was indistinguishable from that of WT mice (Figure 4J). As in the wire clasping experiments, the P110 mice exhibited a motor behavior that was indistinguishable from the 5XFAD control treated mice, the decline in open field exploration in the P110-treated 5XFAD mice is unlikely to reflect a motor deficit induced by P110. Thus, we suggest that this improved behavior is reflective of improved recall in the AD mouse model treated with P110.

## DISCUSSION

Mitochondrial dysfunction, low ATP levels and structural defects are hallmarks of neurodegeneration, in general, and of AD, in particular [24]. Using three different culture models of AD, including human neuroblastoma cells treated with oligomeric Aβ_1–42_, cells overexpressing mutant APP, and cultured AD patient-derived fibroblasts with sporadic and with familial forms of AD, we demonstrated that the mitochondrial structural and function in these AD models is dependent on increased activation of Drp1. Our study demonstrated that increased association of Drp1 with the mitochondria through binding to Fis1 in these AD models leads to excessive mitochondrial fragmentation and dysfunction and to cytotoxicity, as all these defects in the above AD models, including AD patient-derived fibroblasts, were suppressed by treatment with P110. Together, these data suggest that Drp1 increased binding to the mitochondrial adaptor, Fis1, is an important therapeutic target in AD pathology.

P110 inhibitory peptide is composed of a 7-amino-acid peptide, representing a homology sequence between Drp1 and Fis1 [the cargo; [54]], carried across cells and the blood brain barrier with the TAT47–57 carrier peptide [55–58]. We have previously shown that P110 treatment inhibits excessive activation of Drp1 by inhibiting the interaction between Drp1 and Fis1 on the mitochondria under stress conditions in neuronal cells and in models of Parkinson’s disease and Huntington’s disease [36, 58] and that P110 does not affect Drp1 interaction with any other mitochondrial adaptors of Drp1 or with the mitochondrial fusion proteins, Mfn1 or Mfn2. P110 selectively inhibits excessive, but not basal mitochondrial fission and fragmentation [36]. This was demonstrated again in the AD models reported here, where interaction of Drp1/Fis1 was selectively inhibited while the interaction between Drp1 and Mff, Mid49 and Mid51 were not increased in the AD model and P110 did not affect these interactions.

Although the contribution of Aβ oligomers to AD pathogenesis is still unclear, suppressing Aβ-induced cytotoxicity has been the focus of much of the drug development effort for AD [59]. Here, we focused on another pathological hallmark of the disease – mitochondrial structure. As we have shown also in this study, vulnerable neurons in AD brain exhibit significant reduction in mitochondrial length, suggesting abnormal mitochondrial dynamics [25]. Mitochondrial dynamics is critical for the maintenance of mitochondrial integrity and function, including energy metabolism, and prevention of ROS generation, and apoptosis [36, 58]. Under physiological conditions, mitochondrial fission is necessary for division and inheritance of the organelles during cell division, release of cytochrome c and other inter-membrane space proteins during apoptosis [60]. Mitochondrial fission also segregates functional form damaged mitochondria, facilitating in the removal of damaged mitochondria through mitophagy/autophagy [61]. However, excessive mitochondrial fission (fragmentation) results in dysfunctional mitochondrial as evidenced by loss of ATP synthesis and accumulation of ROS that ultimately leads to cell death. Drp1, which mediates both normal and pathological mitochondrial fission, is essential for the proper distribution of mitochondria in axons, dendrites and synapses. We found that while the total Drp1 levels remained unaltered, there was a significant increase in the association of Drp1 with the mitochondria in the presence of Aβ. Importantly, inhibiting Drp1-increased association with the mitochondria using P110 attenuated the structural and functional mitochondrial defects caused by Aβ.

Studies in primary neurons from APP mice (Tg2576 line) showed altered mitochondrial activity, including mitochondrial dynamics, mitochondrial morphology and mitochondrial function [27, 62] and transmission electron microscopy revealed small mitochondria with broken cristae in these neurons [63]. Electron microscopy of the N2a cells incubated with Aβ also showed a significantly increased number of defective mitochondria, indicating that Aβ fragments mitochondria [64] and oligomeric Aβ alters mitochondrial function in various cell types [65]. Consistent with those reports, we observed increased mitochondrial fragmentation with decreased mitochondrial size, decreased interconnectivity and decreased length in multiple *in vitro* AD models. These changes in mitochondrial morphology correlated with increased mitochondrial ROS production as well as reduced ATP generation and mitochondrial membrane potential, similar to what others have reported [20, 24, 27]. Importantly, mitochondrial functions, as measured by ATP production, mitochondrial membrane potential and production of reactive oxygen species, were all significantly improved in cells incubated with P110. Consistent with our study, the recent study of Baek *et al.* [33] using another mouse model of AD (heterozygous APPswe/PSN1dE9) showed Aβ-mediated mitochondrial dysfunctions that resulted in memory deficits. In this study, they treated the mice for one month with Mdivi-1 (10 or 40 mg/kg/day orally) and attributed Mdivi-1 effect to inhibition of Drp1. However, the mechanism of action by which Mdivi-1 affects cell survival also remains unclear [34] and a very recent study demonstrated lack of any effect on Drp1. The study of Bordt *et al.* demonstrates that Mdivi-1 benefit is likely attributed to direct inhibition of mitochondrial Complex 1, which results in reduction of mitochondrial ROS production [35]. Regardless of the molecular target of Mdivi-1, that study supports our finding that an important pathology induced by Aβ is due to mitochondrial dysfunction.

Finally, our finding of abnormal mitochondrial functions in fibroblasts of patients with AD indicates that AD is not only a disease of the CNS and that treatments that addresses only the CNS may expose pathologies in other tissues. The advantage of P110 is that it inhibits excessive mitochondrial fission in all tissues where excessive fission occurs [58, 66], but it does not affect basal (physiological) fission even when used in animals for 5 months at a dose of 3 mg/Kg/day (unpublished data), suggesting that P110 may be safe as a long-term treatment. Importantly, the correction of excessive mitochondrial fission and mitochondrial pathology by P110 was observed in fibroblasts from AD patients regardless of the form of AD (sporadic or familial), suggesting that a selective inhibitor Drp1/Fis1 interaction, such as P110, with may have a broad clinical use to halt or slow down the underlying neuronal degenerative process of AD.

## MATERIALS AND METHODS

All chemicals were purchased from Sigma-Aldrich (St Louis, MO) unless stated otherwise. P110 and P110A along with control peptide were purchased from Ontores, Zhejiang, China.

### Mice

All the experiments were in accordance with protocols approved by the Institutional Animal Care and Use Committee of Stanford University and were performed based on the National Institutes of Health Guide for the Care and Use of Laboratory Animals. We used 5XFAD transgenic mice, which overexpress two transgenes bearing five mutations linked to familial AD: human *APP* (Swedish mutation K670N, M671L; Florida mutation I716V; London mutation V717I) and human *presenilin 1* (*PSEN1* M146L, L286V), under transcriptional control of the mouse Thy1 promoter. 5XFAD lines from the B6SJL genetic background were maintained by crossing hemizygous transgenic mice with B6SJL F1 breeders. These mice exhibit AD-related symptoms earlier than other animal models and amyloid deposition starts in the cortex and subiculum at 2 months of age. The animals used in the P110 treatment study were implanted with a 28 days osmotic pump (Alzet) containing TAT_47–57_ carrier control peptide or P110-TAT_47–57_, which delivered to the mice at a rate of 3 mg/Kg/day, as described previously [67]. The first pump was implanted at an average age of 3 months, with the subsequent pump implantations 28 days later until the age of 6 months.

### Cell culture and peptide treatments

Human neuroblastoma SH5YSY cells were maintained in modified Eagle’s medium (50%) and F12 medium (50%) supplemented with 10% (v/v) fetal bovine serum and 1% (v/v) penicillin/streptomycin.

Mouse Neuro2a (N2a) neuroblastoma cells stably overexpressing the human Swedish mutant (K670N, M671L) APP (N2aAPPsw, clone Swe.10) and human WT APP (N2aWT) were a gift of Dr. Gopal Thinakaran (University of Chicago) and maintained in N2a growth medium as described earlier [42].

AD patient fibroblasts (A1:AG04402; A2:AG07377; A3:AG06840; A4:AG09908, A5:AG05810) and fibroblasts of control healthy individuals (H1:AG07123; H2:AG04146) were purchased from Coriell Institute, USA. H3 (HDFA) was purchased from Invitrogen. All fibroblast cultures were maintained in MEM supplemented with 15% (v/v) fetal bovine serum and 1% (v/v) penicillin/streptomycin at 37°C in 5% CO_2_–95% air.

SH5YSY cells were treated with P110, vehicle (TAT_47–57_), P110 inactive analog (Ala _DALPRGT_ -P110), all at 1 μM final concentration, over a 24-hour incubation in the absence or presence of Aβ_42_ (5 µM). N2a cells were treated with P110 at a final concentration of 1µM every 24-hour in serum free media. Similarly, for patient-derived fibroblasts, TAT or P110-TAT peptides were added once daily for the duration of the experiment at 1 μM final concentration. All experiments were carried out in defined serum free media. Cells with fewer than 18 passages were used in all experiments.

### Aβ preparation

Human synthetic Aβ_1–42_ was purchased from Anaspec (Fremont, CA, USA) and prepared according to protocols described elsewhere [68]. Briefly, lyophilized Aβ_1–42_ peptides were dissolved in 1,1,1,3,3,3-Hexafluoro-2-propanol (HFIP) and aliquoted into polypropylene micro-centrifuge tubes. HFIP was evaporated and the resulting peptide films were stored at −80°C. Before use, these peptide films were reconstituted at a concentration of 1 mM in dimethylsulphoxide (DMSO), then subsequently diluted to 100 μM with ice-cold DMEM (phenol red-free) and incubated for 24 h at 4°C to facilitate the formation of oligomers. Resultant peptides were stored at –80°C until use.

### Cell health assays

#### Measurement of mitochondrial ROS production

To determine mitochondrial ROS production, cells were treated with 5 μM MitoSOX^™^ Red mitochondrial superoxide indicator (Invitrogen) for 10 min at 37°C according to manufacturer’s protocol and fluorescence was analyzed with excitation/emission at 510/580 nm using SpectraMax M2e (Molecular devices).

#### Measurement of mitochondrial membrane potential

Cells were treated with 5 μM JC-1 (5,5′,6,6′-tetrachloro-1,1′,3,3′-tetraethylbenzimidazolcarbocyanine iodide) dye (Invitrogen) in HBSS (Hank’s balanced salt solution) together with DAPI (4’,6-Diamidino-2-Phenylindole, Dihydrochloride) (Thermo Fisher) for 20 min at 37°C, and the fluorescence was analyzed with excitation/emission at 485/590 nm for mitochondrial polarization and at 485/525 nm for mitochondrial depolarization using SpectraMax M2e (Molecular devices). The mitochondrial depolarization was indicated by a decrease in ratio of polarization to depolarization.

#### Mitochondrial toxicity and ATP measurements

To evaluate cell viability, apoptosis and cytotoxicity, the ApoTox-Glo^™^ Triplex assay was used according to the manufacturer’s instructions (Promega, Madison, WI, USA). Briefly, cells were plated in 100 μL medium in an opaque-walled, clear-bottomed, 96-well plate (Greiner Bio-One, Stonehouse, UK). At the end of the culturing period, viability/cytotoxicity reagent was added, mixed by orbital shaking (300 r.p.m. for 30 sec) and incubated for 30 min at 37°C. Fluorescence was measured at 400/505 nm and emission to assess cell viability, and 485/520 nm to assess cytotoxicity; and represented as % Control. Then, 100 μL Caspase-Glo 3/7 reagent was added to each well, mixed by orbital shaking (300 r.p.m. for 30 sec) and incubated for 30 min at room temperature. Luminescence was then measured to assess caspase activation as a marker of apoptosis. To evaluate mitochondria toxicity, cellular ATP levels were determined using Mitochondrial ToxGlo™ assay (Promega, Madison, WI, USA). Briefly, after the cytoxocity measurement, ATP Detection Reagent to the 96-well plate containing cell samples and treatments. Luminescence was measured after 5 minutes to assess ATP levels and represented as % untreated control. All measurements were done using SpectraMax M2e (Molecular devices).

### Glutathione assay

The ratio between reduced and oxidized glutathione is a good indicator of oxidative stress in cells. Cells were plated in 96-well plates and allowed to attach overnight. Post treatments, growth media was removed cells were washed with PBS, and total glutathione and GSSG were each assayed in triplicate via GSH/GSSG-Glo^™^ kit (Promega, Madison, WI, USA) following manufacturers instructions using SpectraMax M2e (Molecular devices).

### Citrate synthase activity assay

50 µg of lysate was added to each well of a 96-well plate containing 160 µl assay buffer (60 mM Tris–HCl, pH 7.5, 200 µM Acetyl CoA, and 250 µM DTNB (5,5′-Dithiobis (2-nitrobenzoic acid)). The lysate was incubated for 5 min, and then 20 µl of 2 mM oxaloacetate was added to each well. OD was measured at 412 nm for 30 min using SpectraMax M2e (Molecular devices). The rate of citrate synthase activity was quantified relative to control cells.

### Bioenergetic profiles

OCR was measured in adherent fibroblasts with an XFe24 Extracellular Flux Analyzer (Seahorse Bioscience). Each control and mutant fibroblast cell line was seeded in 5 wells of an XF 24-well cell culture microplate (Seahorse Bioscience) at a density of 30,000 cells/well in 250 μl of DMEM and incubated for 24 h at 37°C under a 5% CO2 atmosphere. The growth medium was replaced with 575 μl of prewarmed bicarbonate-free DMEM, pH 7.3, and cells were incubated at 37°C for 1 h before starting the assay. After baseline measurements of OCR and ECAR, OCR was measured after sequentially adding to each well 75 μl of oligomycin, 75 μl of FCCP, 75 μl of rotenone and 75 μl of antimycin A to reach working concentrations of 1 μM. OCR and ECAR values were normalized to protein content measured by Sulforhodamine (SRB) assay, following a standard protocol. For data analysis, the following parameters were evaluated: basal respiration, measurement before oligomycin injection subtracted by non-mitochondrial respiration (measurement after antimycin A injection); proton leak–linked respiration, measurement after oligomycin injection subtracted by non-mitochondrial respiration; ATP-linked respiration, basal respiration subtracted by proton leak–linked respiration; maximal respiration, measurement after FCCP injection subtracted by non-mitochondrial respiration; spare capacity, maximal respiration subtracted by basal respiration; complex I–linked respiration, basal respiration subtracted by the measurement after rotenone injection; and maximal complex I–linked respiration, maximal respiration subtracted by the measurement after rotenone injection.

### Immunofluorescence

Cells cultured on 8-well chamber slides were washed with cold PBS, fixed in 4% formaldehyde, and permeabilized with 0.1% Triton X-100. After incubation with 2% normal goat serum (to block nonspecific staining), fixed cells were incubated overnight at 4°C with anti-TOM20 (1:500) (Santa Cruz, USA). Cells were washed with PBS and incubated for 60 minutes with FITC-conjugated goat anti-rabbit IgG (1:500 dilution). The cells were then washed gently with PBS and counterstained with Hoechst 33342 (1:10,000 dilution, Molecular Probes) to visualize nuclei. The coverslips were mounted with Slowfade-antifade reagent (Invitrogen), and images were acquired using an All-in-One Fluorescence Microscope BZ-X700 (Keyence) [36, 58].

### Analysis of immunofluorescence images

Parameters of mitochondrial morphology were further quantified with a custom plugin for ImageJ as described before [69, 70]. In brief, TOM-20 stained images were extracted to grayscale, inverted to show mitochondria-specific fluorescence as black pixels. Images were then thresholded to optimally resolve individual mitochondria and the macro was then used to trace mitochondrial outlines using “analyze particles.” The mean area/perimeter ratio was employed as an index of mitochondrial interconnectivity, with inverse circularity used as a measure of mitochondrial elongation.

### Isolation of mitochondria-enriched fraction and lysate preparation

Cells were washed with cold phosphate-buffered saline (PBS) at pH 7.4 and scraped off using mannitol–sucrose (MS) buffer containing 210 mM mannitol, 70 mM sucrose, 5 mM MOPS (3-(N-morpholino)propanesulfonic acid), 1 mM EDTA, and protease inhibitor cocktail, pH 7.4. The collected cells were passed through a 27-gauge ½-inch needle for lysis, followed by centrifugation at 800 × g to pellet nuclei. The post-nuclear supernatant was further centrifuged at 10,000 × g for 20 min to collect a mitochondria-enriched fraction as previously described before [36, 58].

### Cross-linking immunoprecipitation

Co-immunoprecipitation experiments in patient-derived fibroblasts were carried out as described before [36]. In brief, proteins were cross-linked by incubating the cells in PBS buffer containing 1% formaldehyde and reaction terminated by washing in PBS containing 100 mM glycine. After lysing the cells in PBS containing 1% Triton X-100 and protease inhibitor cocktail, cell suspensions were sonicated and centrifuged to remove insoluble debris. The resultant supernatants were incubated with the antibody and Dynabeads^®^ A/G (Thermo Fischer Scientific, USA) overnight at 4°C and washed in PBS containing 1% Triton X-100 followed by PBS. The immunoprecipitates were then washed, resuspended in the sample buffer, and boiled at 95°C to reverse cross-linking [71].

### Western blot analysis

Protein concentrations were determined using the Bradford assay (Pierce/Thermo Scientific). Proteins were resuspended in Laemmli buffer containing 2-mercaptoethanol, loaded on SDS/PAGE gel and transferred on to nitrocellulose membrane, 0.45 µm (Bio-Rad) as before [36, 66]. Membranes were probed with the indicated antibody and then visualized by ECL (0.225 mM p-Coumaric acid (Sigma), 1.25 mM 3-Aminophtalhydrazide (Luminol) (Fluka) in 1 M Tris pH 8.5). Scanned images of the exposed x-ray film were analyzed with ImageJ to determine relative band intensity. Quantification was performed on samples from independent cultures for each condition. The antibodies used in this study are listed in Supplementary Table 1.

### Hydrogen peroxide assay

The rate of hydrogen peroxide production by fresh isolated mitochondria was determined by the AmplexRed Hydrogen Peroxide/Peroxidase Assay kit (Invitrogen) following the manufacturer’s instructions.

### Lipid peroxidation

Lipid peroxides in brain mitochondria were measured using the leucomethylene blue assay, using tert-butyl hydroperoxide as a standard, by monitoring the 650 nm absorbance after 1 h incubation at RT. The aldehyde product or termination production of lipid peroxidation in brain mitochondria was determined by measuring thiobarbituric acid reactive substances (TBARS). Samples were mixed with 0.15 M phosphoric acid. After the addition of thiobarbituric acid, the reaction mixture was heated to 100°C for 1 h. After cooling and centrifugation, the formation of TBARS was determined by the absorbance of the chromophore (pink dye) at 531 nm using 600 nm as the reference wavelength.

### Aβ ELISA

Aβ levels in mitochondrial fractions and brain cortex were measured by using human Aβ_40_ and Aβ_42_ ELISA kits (Invitrogen) following the manufacturer’s instructions.

### Mitochondrial ATP assay

Aliquots of mitochondria were analyzed by using the ATP Luminescent assay kit (Abcam). Mitochondria were energized with 5 mM glutamate/malate and ATP synthesis was induced with the injection of 500 μM ADP. ATP determination was measured following the manufacturer’s instructions.

### Mouse behavior

#### Activity chamber

The Activity Chamber was used to determine general activity levels, gross locomotor activity, and exploration habits in rodents [67]. Assessment took place in an Open Field Activity Arena (Med Associates Inc., St. Albans, VT. Model ENV-515) mounted with three planes of infrared detectors, within a specially designed sound-attenuating chamber (Med Associates Inc., St. Albans, VT. MED-017M-027). The arena is 43cm(L) × 43 cm (W) × 30 cm(H) and the sound attenuating chamber is 74 cm(L) × 60 cm(W) × 60 cm(H). The animal was placed in the corner of the testing arena, habituated for 10 minutes and allowed to explore the arena for 10 minutes while being tracked by an automated tracking system. A total distance travelled was then measured at baseline and the measurements were repeated after 4 h (recall) and 24 h (re-recall).

### Nest building

Nest-building behavior of mice was tested as described previously [52]. Mice were individually housed for at least 24 h in clean plastic cages with ∼1 cm of corn cob bedding lining the floor and identification cards coded to render the experimenter blind to the sex and genotype of each mouse. Two hours before the onset of the dark phase of the lighting cycle, each cage was supplied a commercially available compressed cotton square (Nestlet, 5 × 5 cm, Ancare, Bellmore, NY, USA). The next morning (∼16 h later), cages were inspected for nest construction. Pictures were taken before evaluation for documentation. Nest construction was scored using the established system of Deacon with a 5-point scale.35 In brief, the scores were as follows: (1) Nestlet not noticeably touched (>90% intact); (2) Nestlet partially torn (50–90% remaining intact); (3) Nestlet mostly shredded but often no identifiable nest site; (4) an identifiable but flat nest; (5) a (near) perfect nest with clear crater (please see the protocol by Deacon for more detailed scoring standard).

### Wire hang test

Mice were subjected to the wire hang test once at 6-month old [53]. They were allowed to grasp, using all limbs, a threaded metal rod (diameter 0.4 cm) that was positioned horizontally 6 inches above their home cage. Mice received three trials (ITI ∼5 min) during which they were allowed to hang until their grip failed or 60 s had elapsed. Latency to fall was recorded and the trial scores were averaged.

### Clasping performance

Clasping behavior was tested in 6-month old mice: mice were suspended by the tail for 60 s and the latency for the hind limbs or all four paws to clasp for at least 3 s was recorded [72].

### Statistical methods

All data are expressed as means ± S.D. Results were collected from three or more independent experiments in at least duplicates. Statistical analysis was performed using Student’s *t-*test, and one- or two-way analysis of variance (ANOVA), as appropriate, with Tukey post hoc tests or Bonferroni post hoc tests. A *p*-value equals to or less than 0.05 was considered significant.

## SUPPLEMENTARY MATERIALS TABLE


