# Supplementary material for: Drp1/Fis1 interaction mediates mitochondrial dysfunction, bioenergetic failure and cognitive decline in Alzheimer's disease
Source: Oncotarget. 2017 Dec 22;9(5):6128–43. doi: 10.18632/oncotarget.23640 (PMC5814200; doi:10.18632/oncotarget.23640)
Supplement: Supplementary file 1 [file oncotarget-09-6128-s001.pdf]

Drp1/Fis1 interaction mediates mitochondrial dysfunction, bioenergetic failure and cognitive decline in Alzheimer's disease

SUPPLEMENTARY MATERIALS

Supplementary Table 1: List of antibodies used in the study

Antibody	Source	Dilution	Catalog no.
Anti-Drp1	BD Transduction Laboratories™	1:1000	611113
Anti-Mff	Proteintech	1:1000	17090-1-AP
Anti-Mid49	Proteintech	1:1000	16413-1-AP
Anti-Mid51	Proteintech	1:1000	20164-1-AP
Anti-Fis1	Proteintech	1:1000	10956-1-AP
Anti-cytochrome C	BD Pharmingen™	1:1500	556432
Anti-VDAC	Abcam	1:2000	14734
Anti-β-actin	Cell Signaling Technology	1:2000	3700
Anti-TOM20	Santa Cruz Biotechnology	1:1000	sc-11415
Anti-TIM17	Santa Cruz Biotechnology	1:500	sc-271152
Anti-Enolase	Santa Cruz Biotechnology	1:1500	sc-15343
Anti-phospho Drp1 ser 637	Cell Signaling Technology	1:500	3455
Anti-phospho Drp1 ser 617	Cell Signaling Technology	1:500	4867
Anti-p62	Abcam	1:1000	56416
Anti-Beclin-1	Cell Signaling Technology	1:1000	3738
Anti-ATG5	Cell Signaling Technology	1:500	2630
Anti-ATG3	Cell Signaling Technology	1:500	3415
Anti-LC3BII	Cell Signaling Technology	1:1000	3868
Anti-LAMP1	Cell Signaling Technology	1:500	9091
Anti-CathB	Abcam	1:500	ab58802
Anti-CathD	Abcam	1:500	ab75852
Anti-cleaved caspase 3	Cell Signaling Technology	1:250	9661
Anti-phosho JNK	Cell Signaling Technology	1:500	9252
Anti FL-APP	Cell Signaling Technology	1:1000	2450
anti-β-Amyloid Precursor Protein	Invitrogen	1:1000	51-2700
Anti-BACE1	Cell Signaling Technology	1:1000	5606
Anti-Mfn2	Proteintech	1:500	12186-1-AP
Anti-Opa1	Santa Cruz Biotechnology	1:500	sc-393296
